# Prognostic determinants in cancer survival: a multidimensional evaluation of clinical and genetic factors across 10 cancer types in the participants of Genomics England’s 100,000 Genomes Project

**DOI:** 10.1007/s12672-024-01310-8

**Published:** 2024-09-15

**Authors:** Jurgita Gammall, Alvina G. Lai

**Affiliations:** 1 Institute of Health Informatics, University College London, 222 Euston Road, London, NW1 2DA UK; 2 Oracle Global Services Limited, London, UK

**Keywords:** Cancer, Prognosis, Survival, Factors, Genomics, Electronic health records

## Abstract

**Background:**

Cancer is a complex disease, caused and impacted by a combination of genetic, demographic, clinical, environmental and lifestyle factors. Analysis of cancer characteristics, risk factors, treatment options and the heterogeneity across cancer types has been the focus of medical research for years. The aim of this study is to describe and summarise genetic, clinicopathological, behavioural and demographic characteristics and their differences across ten common cancer types and evaluate their impact on overall survival outcomes.

**Methods:**

This study included data from 9977 patients with bladder, breast, colorectal, endometrial, glioma, leukaemia, lung, ovarian, prostate, and renal cancers. Genetic data collected through the 100,000 Genomes Project was linked with clinical and demographic data provided by the National Cancer Registration and Analysis Service (NCRAS), Hospital Episode Statistics (HES) and Office for National Statistics (ONS). Descriptive and Kaplan Meier survival analyses were performed to visualise similarities and differences across cancer types. Cox proportional hazards regression models were applied to identify statistically significant prognostic factor associations with overall survival.

**Results:**

161 clinical and 124 genetic factors were evaluated for prognostic association with overall survival. Of these, 116 unique factors were found to have significant prognostic effect for overall survival across ten cancer types when adjusted for age, sex and stage. The findings confirmed prognostic associations with overall survival identified in previous studies in factors such as multimorbidity, tumour mutational burden, and mutations in genes BRAF, CDH1, NF1, NRAS, PIK3CA, PTEN, TP53. The results also identified new prognostic associations with overall survival in factors such as mental health conditions, female health-related conditions, previous hospital encounters and mutations in genes FANCE, FBXW7, GATA3, MSH6, PTPN11, RB1, RNF43.

**Conclusion:**

This study provides a comprehensive view of clinicopathological and genetic prognostic factors across different cancer types and draws attention to less commonly known factors which might help produce more precise prognosis and survival estimates. The results from this study contribute to the understanding of cancer disease and could be used by researchers to develop complex prognostic models, which in turn could help predict cancer prognosis more accurately and improve patient outcomes.

**Supplementary Information:**

The online version contains supplementary material available at 10.1007/s12672-024-01310-8.

## Background

Cancer is a leading cause of death, accounting for nearly one in six deaths worldwide [1]. It is a complex disease, caused and impacted by a combination of genetic, demographic, clinical, environmental and lifestyle factors. Cancer is also a very heterogenous disease with more than 100 distinct types that differ in their symptoms, severity, treatment and survival [2]. Analysis of cancer characteristics, risk factors, treatment options and survival has been at the forefront of medical research and health care practice for decades.

In a previous systematic review we conducted [3], we assessed the published evidence on clinicopathological factors and genetic mutations associated with prognosis across 11 types of cancer. Our review, which incorporated 247 articles in full text after reviewing 2824 articles by title and abstract, and included a pan-cancer investigation, identified a set of biomarkers that could potentially be used to tailor treatments according to patients’ unique genetic and clinical characteristics.

Numerous previous studies analysed genetic mutations associated with cancer to understand and explain the risk factors for cancer development, survival and inform personalised cancer treatments [4–8]. Mutations in genes such as TP53, BRCA1, BRCA2, BRAF and KRAS have been widely reported to have a significant prognostic effect in multiple cancer types including breast, colorectal, lung, ovarian, prostate and other cancers. Generally, tumour mutational burden (TMB) has been found to be associated with cancer survival on pan-cancer level.

There are also extensive resources available on the association between cancer and demographic (e.g., age), environmental (e.g., radiation), biological (e.g., viral infection) and lifestyle (e.g., smoking) factors [9–12]. Demographic factors such as age, gender and ethnicity are widely known to be associated with cancer survival. Lifestyle factors such as smoking, alcohol use and weight have been identified as risk factors for shorter overall survival. Certain comorbidities such as diabetes, chronic obstructive pulmonary disease (COPD) and anaemia have been reported to have an association with cancer survival.

Building upon our initial systematic review, we recognised a critical gap in the understanding of the interplay between genetic and clinical factors on a pan-cancer level. While the systematic review provided a comprehensive knowledge base of biomarkers, the existing literature largely consisted of studies focusing on a single type of cancer. There are only a few studies that have linked both genetic and clinical data of cancer patients to analyse prognostic effects of biomarkers, which have shown that investigating genetic and clinical interactions helps improve the understanding of cancer disease [13–15]. Yet, this area remains unexplored on pan-cancer level and across different types of health care data.

To address this knowledge gap, this study leverages data available through the 100,000 Genomes Project led by Genomics England [16], linked with Hospital Episode Statistics (HES) data [17], National Cancer Registration and Analysis Service (NCRAS) data [18] and Office of National Statistics (ONS) data [19]. This unique dataset enables a multidimensional analysis on thousands of patients representing a broad spectrum of cancer types, thereby deepening our understanding of the interplay between genetic, clinicopathological and demographic factors in cancer.

The primary aim of this study is to describe and summarise the salient genetic, clinicopathological, behavioural and demographic characteristics across ten common cancer types. Furthermore, we seek to examine the variability in cancer treatments and survival outcomes across these cancers. In line with this aim, the specific objectives are to: (1) characterise the genetic mutations prevalent in ten common cancer types and assess their prognostic effect on survival, (2) summarise the key demographic and behavioural risk factors associated with these cancers and their survival outcomes, (3) characterise the prevalence of clinical conditions and multimorbidity in these cancers and their prognostic effect on overall survival, (4) assess the variability in treatment strategies and their impact on survival outcomes across these cancers. By offering a comprehensive analysis of the complexities of cancer, this study seeks to fill a critical gap in our understanding and foster progress in cancer research and treatment.

## Methods

### Data sources

Analyses presented in this study were based on five data sources from Genomics England’s Main Programme (v15 updated on 26th May 2022) [20]. Firstly, primary clinical data collected by Genomic Medicine Centres (GMCs) for participants upon enrolment in the 100,000 Genomes Project programme were used to create the patient cohort, classify patients by cancer type and collect demographic information. Data from ONS [19] were used to determine mortality information (date of death and cause of death). The NCRAS data [18] were used for information about participants’ diagnosis, tumour and treatment as well as a secondary source for demographic information. The HES data [17] were used for information about participants’ comorbidities and encounters with hospital care services. The HES data were also used as a third source for participants’ demographic information. Genomics England’s Cancer Tiering data source [21] was used for genetic variant information.

### Cohort selection

**Fig. 1:**
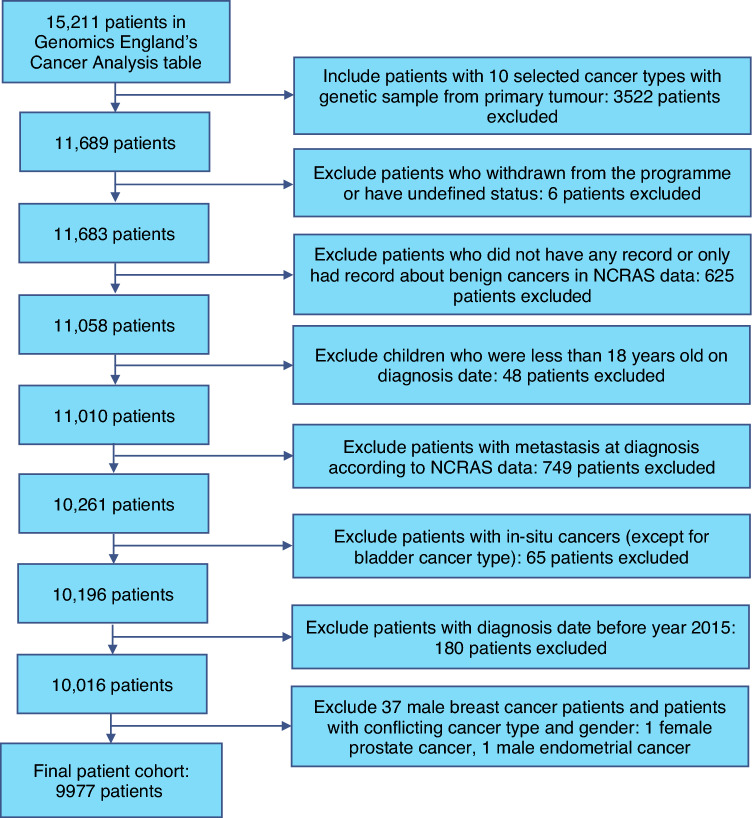
Summary of data exclusions. Flowchart of exclusions applied to the patient cohort included in the study

The patient cohort was created using information provided by Genomics England for participants in the 100,000 Genomes Project who had their tumour samples sequenced, variants called and their data successfully passed quality control. 15,211 patients with cancer were available in the Genomics England database, which was reduced to 11,689 patients after selecting 10 cancer types with most data availability (bladder, breast, colorectal, endometrial, glioma, leukaemia, lung, ovarian, prostate, renal) and only including those patients who had at least one genetic sample taken from the primary tumour. Six patients were excluded from the cohort due to their withdrawal or undefined participation status in the programme. Further 625 patients were excluded who did not have any record or only had a record about benign cancer in the NCRAS data. Children who were less than 18 years old on diagnosis date were also excluded from the cohort (48 patients). Patients with metastasis on diagnosis as well as patients with in-situ cancers (except for bladder cancer) were excluded from the study (749 and 65 patients respectively) in order to maintain comparability in the patient cohort. In-situ bladder cancers are known to be malignant and aggressive, contrary to other in-situ cancer types, and therefore were not excluded from analysis [22]. 180 patients with cancer diagnosis before the year 2015 were excluded, because of large difference between diagnosis date and the date when the genetic sample was taken. Finally, 37 patients with male breast cancer and 2 patients with conflicting gender and diagnosis were excluded from the cohort. 9977 patients were included in the final patient cohort. A summary of all exclusions applied to the patient cohort is provided in Fig. 1.

### Data cleaning and pre-processing

Data from different sources were linked using participant id field provided by Genomics England. In some cases, patients had multiple records in the primary data collected by Genomics England or in other sources. In situations where a patient had information on multiple tumours in the NCRAS data, participant id in combination with histology information were used to link the data. Where a patient had multiple matches using this method, the date difference between diagnosis date in the NCRAS data and tumour delivery date in the Genomics England data was used to identify the correct record. In rare cases, where patients had multiple genetic samples taken from their primary tumour, all samples were kept in the analysis.

Demographic data were populated using three data sources. If available, data were taken from Genomics England primary data sources, then from NCRAS, then from HES. Age at diagnosis was derived using date of birth and date of diagnosis. Ethnicity data were organised into six ethnicity categories according to ONS classification [23]. Deprivation data were based on Index of Multiple Deprivation (IMD) [24].

Cancer sub-type and site classification was cleaned using histology coded data and categories with low patient counts were combined into ‘other’ category. Tumour grade and stage data were taken from the NCRAS data source, cleaned by replacing invalid information with ‘unknown’ category and standardised to meaningful categories.

Referral route data was combined with data about cancer screening programmes. Cancer waiting times data was filtered to only include information on waiting times for the first cancer treatment. To link waiting time data with the right tumour for each patient, date difference between treatment start and diagnosis date was used.

Four treatment types were derived from the treatment data available in NCRAS; chemotherapy, hormone therapy, radiotherapy and immunotherapy. The majority of patients in the cohort had surgery, which was caused by the design of data collection which focused on surgical resection samples (about 92% of all genetic samples were collected from surgical resection). Therefore, a surgery flag was not included in the analysis. Using systemic anti-cancer therapy activity data, eight anti-cancer drug categories were derived: (1) alkylating agents, (2) antimetabolites, (3) antitumour agents, (4) hormone therapy, (5) kinase inhibitors, (6) plant alkaloids, (7) targeted therapy and immunotherapy, and (8) other therapy.

Healthcare utilisation measures were derived using the HES data source, broken down by accident and emergency, inpatient and outpatient care. All the measures were calculated on spell level and over four lookback periods: last 6 months, last 12 months, last 5 years, full history.

Diagnoses and procedures recorded in the HES data on or before diagnosis date were used to determine what conditions (other than cancer) a patient had. CALIBER phenotypes [25] with an addition of chronic kidney disease were used to derive condition flags using 10th revision of the International Classification of Diseases (ICD10) diagnosis codes and Operating Procedure Codes Supplement (OPCS) procedure codes. 302 condition flags were derived in total. Patient counts were calculated and reviewed for each condition, broken down by cancer type. Conditions with very low patient counts (all cancer types having fewer than 10 patients or fewer than 0.5% of patients with that condition) were either excluded or grouped with other conditions where possible. 82 conditions were included in the final list of conditions after groupings and exclusions were applied. Data quality checks and cleaning were applied for gender-specific conditions, namely female genital tract disorders, female infertility, hyperplasia of prostate, infection of male genital system, menstruation disorder.

### Genetic variant filtering

Genomics England’s Cancer Tiering data contains non-synonymous, splice site and ribonucleic acid (RNA) gene single nucleotide variants (SNVs) and small indels found per participant sample. SNVs and small indels were normalised and annotated using Cellbase and ClinVar databases [21]. Germline variants were included in this data set using gene panels [26] for each tumour type. Somatic variants were classified into three domains. Domain 1 included variants in a panel of potentially actionable genes. Domain 2 included variants in a panel of cancer-related genes. Other variants were classified to Domain 3. In the cohort of 9977 patients, 18,965 genes and 3,675,866 variants were recorded in the Cancer Tiering data set.

Multiple variant filtering criteria were applied to identify relevant SNVs and small indels that could help determine prognosis and predict survival in cancer patients. Firstly, a gene-level filter was applied using a selected list of 810 genes. The list was created combining the following two resources:

- Genes identified in a systematic review [3], filtered on 10 relevant cancers
- Cancer-related genes in the COSMIC Census database [27], filtered on 10 relevant cancers

Out of 810 selected genes, 790 were found in the Cancer Tiering data. It is likely that no patients in the cohort had variants in the remaining 20 genes, hence they did not appear in the data. Out of 9977 patients in the cohort, 9918 patients had at least one variant in at least one gene in the selected list. There were 303,748 variants recorded in the patient cohort when filtered on the selected genes. Of these, 18,079 variants were pathogenic or likely pathogenic using ClinVar annotation. 280 genes from the selected list of 790 genes remained in the analysis after filtering on pathogenic or likely pathogenic variants.

Genes with very small patient numbers were excluded (genes that had variants in fewer than 10 patients or frequency of less than 0.5% when grouped by cancer type). The final filtered genetic variant data included 99 genes and 17,041 variants. 24 of 99 genes had both somatic and germline variants, while the rest 75 genes had only somatic variants. 99 binary flags were created to indicate whether a patient had a somatic variant, and 24 binary flags were created to indicate whether a patient had a germline variant in selected genes.

### Survival analysis

Overall and disease-specific Kaplan Meier survival curves were plotted to visualise survival probabilities by cancer type. The results for overall survival were similar to disease-specific survival. Given that the recorded cause of death can often be a complication of cancer, decease-specific survival would underestimate mortality. Therefore, overall survival was used as the end point in all survival analyses.

To identify demographic, clinical and genetic factors that are important predictors for overall survival, multiple Cox proportional hazards regression models were run. A model for each cancer type and factor combination in the dataset was created, adjusted for age, sex and stage where appropriate for cancer type. All numeric factors were converted to categorical based on the data distributions. Factors with large variation across cancer types such as TMB or waiting times were converted into categories using percentiles, calculated for each cancer type separately. Missing data were categorised to ‘Unknown’ category.

Important prognostic factors for overall survival were identified using p-value < 0.05 after adjusting for small patient numbers. A factor was tested in Cox proportional hazards regression model only when for a selected cancer type, there were at least 10 patients in each stratum of the factor. Significant results from Cox proportional hazards regression models are provided in the Results section. Proportional hazards assumption was tested and met for all models.

## Results

### Patient cohort

9977 patients with 10 cancer types were included in this study. Breast and colorectal cancer types had the largest number of patients (2605 and 2247 respectively), and bladder and leukaemia cancer types had the smallest number of patients (353 and 238 respectively, Table 1). Mortality rates ranged from 2.8% for patients with prostate cancer to 64.3% for patients with glioma cancer. Patients with bladder, colorectal and lung cancers were older on average compared to other cancer types, while glioma and leukaemia cancer types had the youngest patients. All cancer types that were not sex-specific, had a higher percentage of male patients compared to female patients ranging from 52.0% in lung cancer to 70.8% in bladder cancer. Patients with white ethnic background constituted the majority of patients in all cancer types ranging from 84.5% in breast cancer to 94.6% in lung cancer. Patients with most cancer types were reasonably equally spread across deprivation quintiles, with a slightly higher proportion in affluent areas. Lung cancer was the only exception where the highest proportion of patients (24.5%) were from the most deprived areas.

**Table 1:** Patient cohort summary

Total patients	Bladder		Breast		Colorectal		Endometrial		Glioma		Leukaemia		Lung		Ovarian		Prostate		Renal	
	353		2605		2247		762		434		238		1317		436		462		1123	
*Status*																				
Alive	254	71.95%	2324	89.21%	1788	79.57%	608	79.79%	155	35.71%	151	63.45%	817	62.03%	284	65.14%	449	97.19%	997	88.78%
Deceased	99	28.05%	281	10.79%	459	20.43%	154	20.21%	279	64.29%	87	36.55%	500	37.97%	152	34.86%	13	2.81%	126	11.22%
*Age group*																				
18–49	16	4.53%	579	22.23%	152	6.76%	38	4.99%	152	35.02%	74	31.09%	40	3.04%	62	14.22%	10	2.16%	153	13.62%
50–69	117	33.14%	1239	47.56%	955	42.50%	408	53.54%	210	48.39%	114	47.90%	599	45.48%	254	58.26%	368	79.65%	629	56.01%
70 +	220	62.32%	787	30.21%	1140	50.73%	316	41.47%	72	16.59%	50	21.01%	678	51.48%	120	27.52%	84	18.18%	341	30.37%
*Sex*																				
Female	103	29.18%	2605	100.00%	903	40.19%	762	100.00%	160	36.87%	91	38.24%	632	47.99%	436	100.00%	0	0.00%	426	37.93%
Male	250	70.82%	0	0.00%	1344	59.81%	0	0.00%	274	63.13%	147	61.76%	685	52.01%	0	0.00%	462	100.00%	697	62.07%
*Ethnicity*																				
White	328	92.92%	2202	84.53%	2025	90.12%	651	85.43%	399	91.94%	211	88.66%	1246	94.61%	392	89.91%	362	78.35%	1012	90.12%
Asian	9	2.55%	127	4.88%	73	3.25%	39	5.12%	14	3.23%	0	0.00%	14	1.06%	16	3.67%	11	2.38%	33	2.94%
Black	9	2.55%	126	4.84%	49	2.18%	20	2.62%	0	0.00%	6	2.52%	20	1.52%	5	1.15%	50	10.82%	19	1.69%
Other/Unknown	7	1.98%	150	5.76%	100	4.45%	52	6.82%	21	4.84%	21	8.82%	37	2.81%	23	5.28%	39	8.44%	59	5.25%
*Deprivation*																				
1—Least deprived	62	17.56%	514	19.73%	518	23.05%	169	22.18%	103	23.73%	53	22.27%	240	18.22%	127	29.13%	114	24.68%	232	20.66%
2—Second least deprived	68	19.26%	514	19.73%	493	21.94%	169	22.18%	101	23.27%	47	19.75%	264	20.05%	103	23.62%	103	22.29%	239	21.28%
3—Third most deprived	84	23.80%	510	19.58%	464	20.65%	154	20.21%	96	22.12%	60	25.21%	240	18.22%	95	21.79%	86	18.61%	272	24.22%
4—Second most deprived	72	20.40%	560	21.50%	435	19.36%	154	20.21%	66	15.21%	47	19.75%	249	18.91%	71	16.28%	96	20.78%	208	18.52%
5—Most deprived	66	18.70%	486	18.66%	330	14.69%	116	15.22%	68	15.67%	31	13.03%	323	24.53%	40	9.17%	60	12.99%	171	15.23%

Table provides censoring status and demographic information about the patient cohort included in the study such as age, sex, ethnicity and deprivation by cancer type

### Tumour

**Fig. 2:**
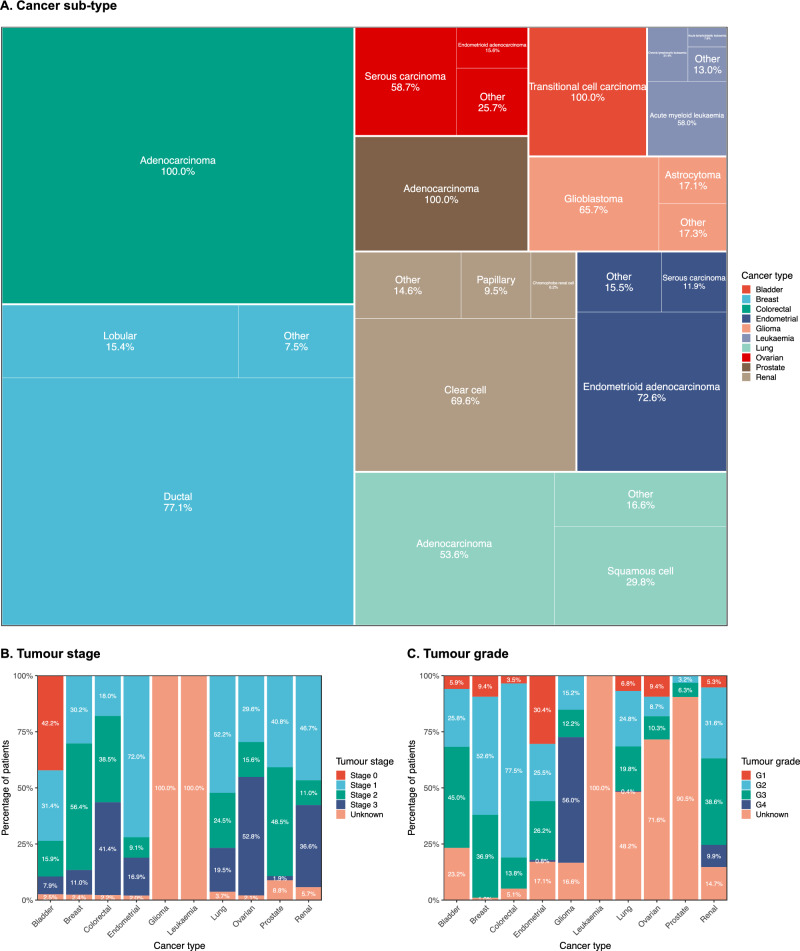
Tumour characteristics. **A** Cancer sub-type. Percentage of patients in cancer sub-type category out of total patients by cancer type. Percentages sum up to 100% for each cancer type. Box sizes represent the patient counts in the whole patient cohort. For cancers that did not have classification to different sub-types, one box with 100% is included. **B** Tumour stage. Percentage of patients by tumour stage compared across cancer types. For cancers that did not have classification by stage, 100% unknown is shown in the chart. **C** Tumour grade. Percentage of patients by tumour grade compared across cancer types. Patients with leukaemia did not have information about grade and therefore shown as 100% unknown in the chart

The majority of patients with breast cancer had ductal carcinoma (77.1%), endometrial cancer—endometrioid adenocarcinoma (72.6%), glioma—glioblastoma (65.7%), leukaemia—acute myeloid leukaemia (58.0%), lung cancer—adenocarcinoma (53.6%), ovarian cancer—serous carcinoma (58.7%), renal cancer—clear cell carcinoma (69.6%). Bladder, colorectal and prostate cancers did not have meaningful information about cancer sub-types available in the data (Fig. 2, Part A). Information about tumour sites is provided in supporting materials (Additional file 1).

The highest proportion of patients in the cohort had Stage 1 cancer (33.8%) followed by Stage 2 (31.7%), with large variation across cancer types (Fig. 2, Part B). Endometrial and bladder cancers had the highest proportion of patients with stages 0 and 1 (73.6% and 72.0% respectively). Breast and prostate cancers had the highest proportion of patients with Stage 2 tumour (56.4% and 48.5% respectively). Colorectal, ovarian, and renal cancers had the highest proportion of patients with Stage 3 tumour (41.4%, 52.8% and 36.6% respectively). Endometrial cancer had the highest proportion of grade 1 tumours (30.4%), while glioma had the highest proportion of grade 4 tumours (56.0%). The majority of patients had grade 2 or 3 tumours in all other cancer types where grade information was available (Fig. 2, Part C).

### Treatment

**Fig. 3:**
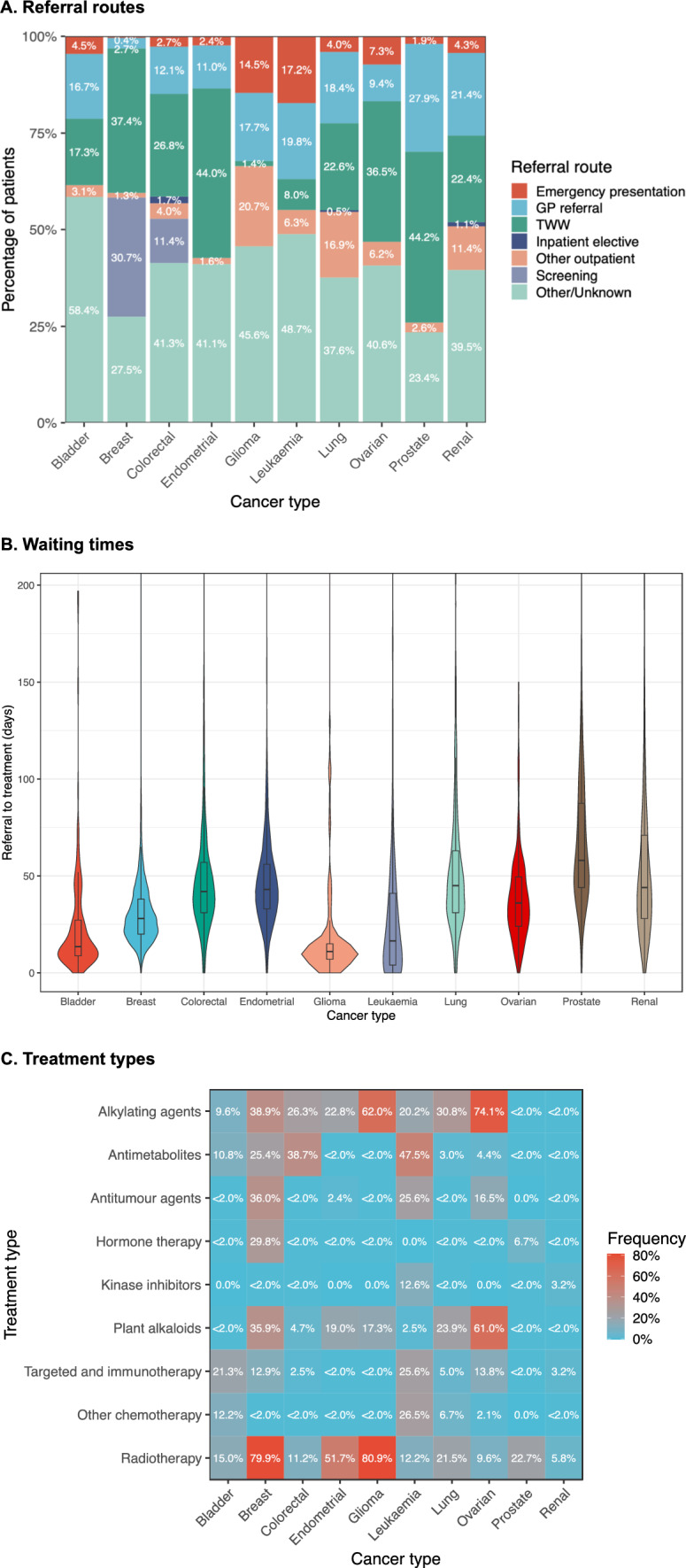
Referral and treatment. **A** Referral routes. Percentage of patients by referral route compared across cancer types. **B** Waiting times. Violin plot demonstrates distribution of days from referral to treatment by cancer type. **C** Treatment types. Heatmap demonstrates frequencies of treatment types compared across cancer types. Frequencies are calculated as percentage of patients who were treated with each therapy type out of total patients in cancer type. To avoid patient identification, percentages lower than 2% that could imply small patient counts were combined into one category and presented as “ < 2.0%”

Majority of patients in all cancer types except glioma had GP referral or two-week wait as their referral route (Fig. 3, Part A). Breast and colorectal cancers had some patients referred through national screening programmes (30.7% and 11.4% respectively). Leukaemia and glioma cancers had the highest proportion of patients referred though emergency presentation of all cancers (17.2% and 14.5% respectively). Shortest referral to treatment waiting times were observed for glioma, leukaemia and bladder cancers (11, 16.5 and 13.5 median days respectively), while longest waiting times were for prostate cancer (58 median days, Fig. 3, Part B).

Alkylating agents were the most common chemotherapy treatment across different cancer types with ovarian and glioma cancers having the largest proportion of patients who had this treatment (74.1% and 62.0% respectively, Fig. 3, Part C). Plant alkaloids were also a popular treatment with ovarian and breast cancers having the largest proportion of patients with this treatment (61.0% and 35.9% respectively). Antimetabolites were the most common chemotherapy treatment in leukaemia (47.5%) and colorectal (38.7%) cancers. Hormone therapy was mainly applied in patients with breast (29.8%) and prostate (6.7%) cancers. Kinase inhibitors were most popular in leukaemia (12.6%). Targeted therapy and immunotherapy were most popular in leukaemia (25.6%) and bladder (21.3%) cancers. Patients with all types of cancer were treated with radiotherapy ranging from 5.8% of patients with renal cancer to 80.9% of patients with glioma cancer. Lowest levels of chemotherapy cumulative dose were observed for glioma, lung and endometrial cancers, while highest levels were observed for leukaemia, colorectal and breast cancers. Highest levels of teletherapy fields were observed in breast, leukaemia and colorectal cancers, while highest levels of radiography dose were observed in breast, prostate, glioma and lung cancers. Information about chemotherapy and radiotherapy dosage is available in supporting materials (Additional file 2).

### Medical history

**Fig. 4:**
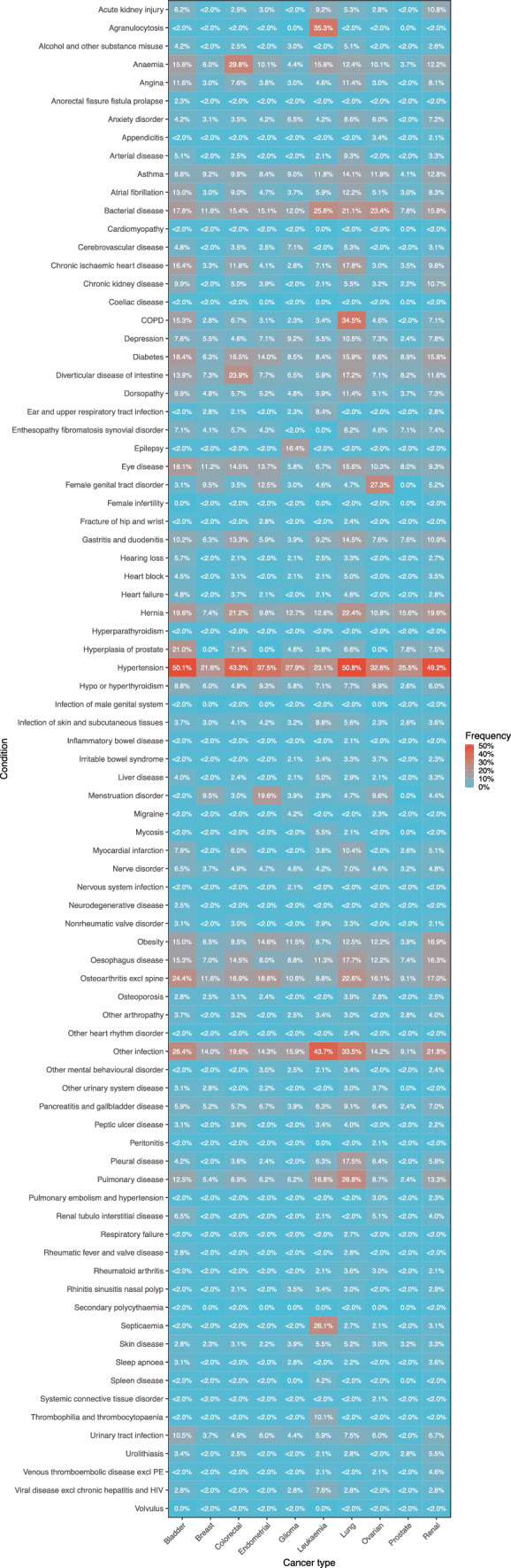
Condition frequencies. Heatmap demonstrates frequencies of selected conditions compared across cancer types. Frequencies are calculated as percentage of patients who had the condition out of total patients in cancer type. To avoid patient identification, percentages lower than 2% that could imply small patient counts were combined into one category and presented as “ < 2.0%”

Hypertension was the most common pre-existing condition in all cancers except leukaemia, ranging from 21.6% in breast cancer to 50.8% in lung cancer (Fig. 4). The most common condition observed in patients with leukaemia was other infection (43.7%), which was also prevalent in other cancer types. Anaemia was common in patients with a few cancer types with the highest rate in colorectal cancer (29.8%). Heart diseases such as angina, atrial fibrillation, and chronic ischaemic heart disease were most prevalent in bladder and lung cancers. Female health-related conditions where prevalent in breast, endometrial and ovarian cancers with 27.3% of ovarian cancer patients having female genital tract disorder and 19.6% of endometrial cancer patients having menstruation disorder. A high percentage of patients with bladder cancer had osteoarthritis (24.4%) and hyperplasia of prostate (21.0%). A lot of patients with colorectal cancer had diverticular disease of intestine (23.9%) and hernia (21.2%). Patients with glioma had high prevalence in epilepsy (16.4%). Patients with leukaemia had high prevalence in agranulocytosis (35.3%) and septicaemia (26.1%). Lung cancer patients had high prevalence in COPD (34.5%) and pulmonary disease (26.8%).

Patients with renal cancer had the highest number of previous hospital admissions over longer periods of time (full history and last 5 years), while patients with glioma cancer had the highest number over shorter periods of time (last 12 months and last 6 months). Patients with lung and bladder cancers also had high number of previous hospital admissions compared to other cancer types over longer periods of time, which could be partly explained by a higher proportion of older patients in those cancer types. Patients with glioma and leukaemia had the highest number of previous emergency admissions across all time periods. The lowest numbers of previous emergency and all admissions were observed for patients with breast, endometrial and prostate cancers across all time periods. Information about previous hospital admissions is available in supporting materials (Additional file 3).

### Genetic mutation summary

**Fig. 5:**
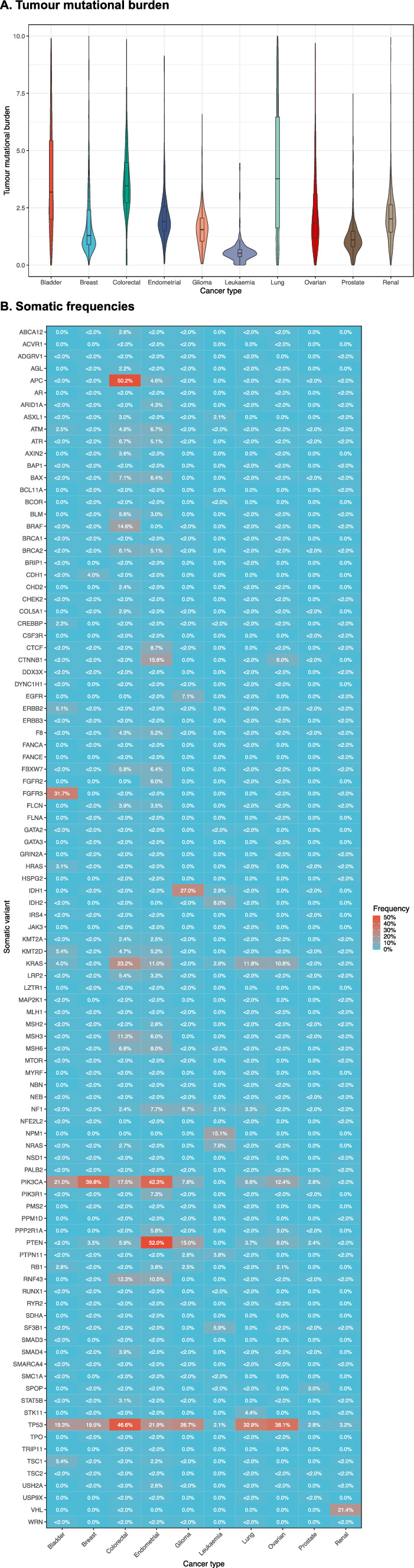
TMB and somatic frequencies. **A** Tumour mutational burden. Violin plot demonstrates distribution of tumour mutational burden measured as number of somatic coding variants per mb by cancer type. **B** Somatic frequencies. Heatmap demonstrates frequencies of somatic mutations in selected genes compared across cancer types. Frequencies are calculated as percentage of patients who had the mutation out of total patients in cancer type. To avoid patient identification, percentages lower than 2% that could imply small patient counts were combined into one category and presented as “ < 2.0%”

The highest median tumour mutational burden (measured as number of somatic coding variants per mb) was observed in lung, colorectal and bladder cancers, which also showed the highest variation among patients (Fig. 5, Part A). The lowest median tumour mutational burden was observed for patients with leukaemia, prostate and breast cancers.

Germline variants were very rare in the patient cohort. The only two genes with more than 2% of patients having germline mutations were BRCA1 and BRCA2 in ovarian cancer (5.3% and 2.8% respectively). Germline variant frequencies are provided in supporting materials (Additional file 4). The gene with the largest number of somatic variants identified was TP53, ranging from only 2.1% of patients with the variant in leukaemia to 46.6% of patients with the variant in colorectal cancer (Fig. 5, Part B). PIK3CA gene also had a large percentage of patients with somatic variants in multiple cancer types such as bladder (21.0%), breast (39.8%), colorectal (17.5%), endometrial (42.3%), glioma (7.8%), lung (6.6%) and ovarian (12.4%).

31.7% of patients with bladder cancer had a somatic variant in FGFR3 gene. Patients with colorectal cancer had large frequency of variants in genes APC (50.2%), KRAS (23.2%), BRAF (14.6%), RNF43 (12.3%), and MSH3 (11.3%). Patients with endometrial, lung and ovarian cancers also had a large frequency of variants in KRAS (11.0%, 11.8% and 10.8% respectively). Patients with endometrial cancer also had high prevalence of variants in PTEN (52.0%), CTNNB1 (15.6%), and RNF43 (10.5%) genes. Somatic variants in PTEN gene were also prevalent in patients with glioma (15.0%), as well as variants in IDH1 (27.0%). Patients with leukaemia had low prevalence of variants in most genes, even the most common ones such as TP53 and PIK3CA, but had 15.1% of patients with a variant in NPM1 gene. Patients with renal cancer had high prevalence of somatic variants in VHL gene (21.4%).

### Kaplan Meier survival analysis

**Fig. 6:**
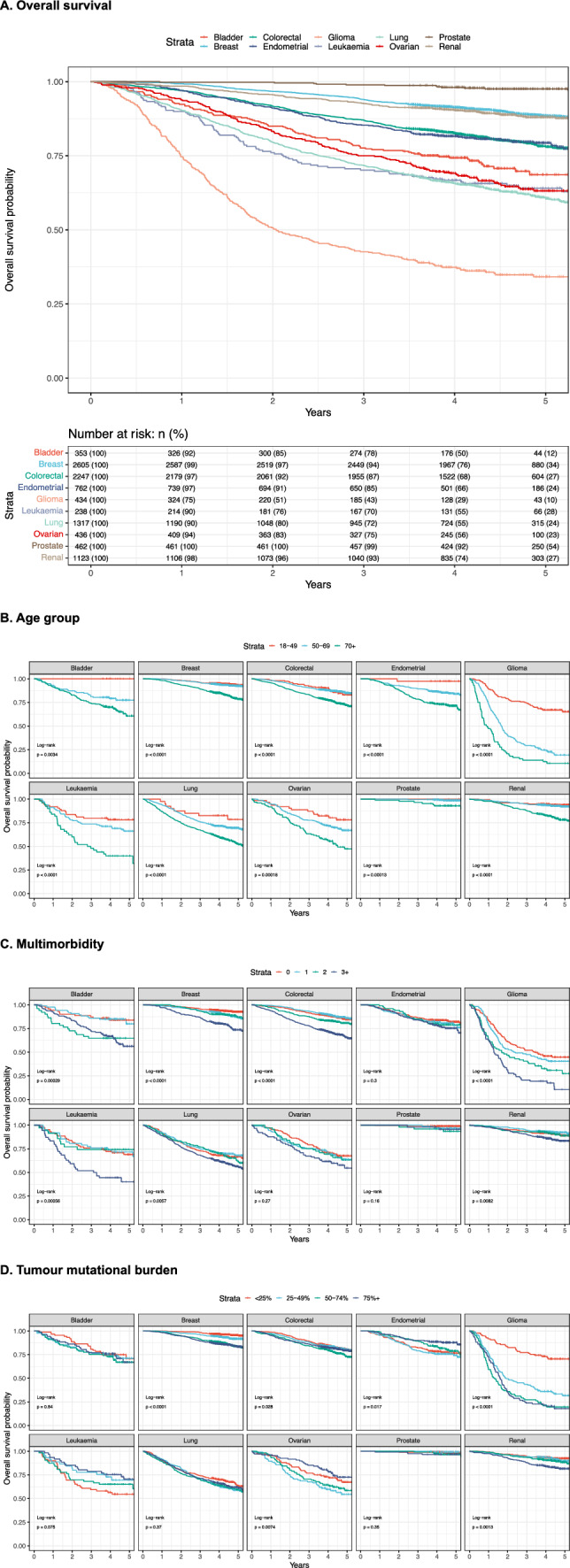
Kaplan Meier survival curves. **A** Overall survival. Kaplan Meier survival curves demonstrate overall survival probability over 5 years by cancer type. Numbers at risk demonstrate patient counts with percentage of total patients in cancer type provided in brackets. **B** Age group. Kaplan Meier survival curves demonstrate overall survival probability by age group faceted by cancer type including log-rank test p-value. **C** Multimorbidity. Kaplan Meier survival curves demonstrate overall survival probability by number of long-term conditions faceted by cancer type including log-rank test p-value. **D** Tumour mutational burden. Kaplan Meier survival curves demonstrate overall survival probability by tumour mutational burden percentiles faceted by cancer type including log-rank test p-value

Patients with glioma had the lowest overall survival probabilities compared to other cancer types, with less than 50% probability of surviving 3 years and less than 40% probability of surviving 5 years (Fig. 6, Part A). Other cancer types had between 60 to 95% probability of surviving 5 years. Prostate cancer had the highest survival probability of about 95% for surviving at least 5 years while breast and renal cancers had a probability of over 85%. Similar results were found for disease-specific survival (Additional file 5 in supporting materials).

Older patients had significantly lower survival probabilities in all cancer types compared to younger patients (Fig. 6, Part B). The most extreme effect of age on overall survival was observed in glioma where the youngest age group of 18–49 year olds had 5-year overall survival probability of around 63% while the oldest age group of 70 year olds and over had 5-year overall survival probability of around 12%.

Patients with a higher number of long-term conditions had lower probabilities for overall survival in all cancer types (Fig. 6, Part C). The largest differences in survival probabilities by multimorbidity categories were observed in glioma and leukaemia. Leukaemia patients with none to two comorbidities had a probability of around 70–75% for 5-year overall survival, while patients with three or more comorbidities had a probability of around 38%. Glioma patients with no comorbidities had a probability of around 45% for 5-year overall survival, while patients with three or more comorbidities had a probability of around 12%.

TMB was found to be an important prognostic factor for overall survival in multiple cancer types. In glioma and breast cancers, higher TMB was associated with shorter 5-year overall survival, while the opposite was found for leukaemia and endometrial cancers (Fig. 6, Part D). The largest effect was observed for glioma, where patients in the lowest TMB category had a 5-year overall survival probability of around 70%, while patients in the highest category had a probability of around 15%.

### Cox proportional hazards survival analysis

**Fig. 7:**
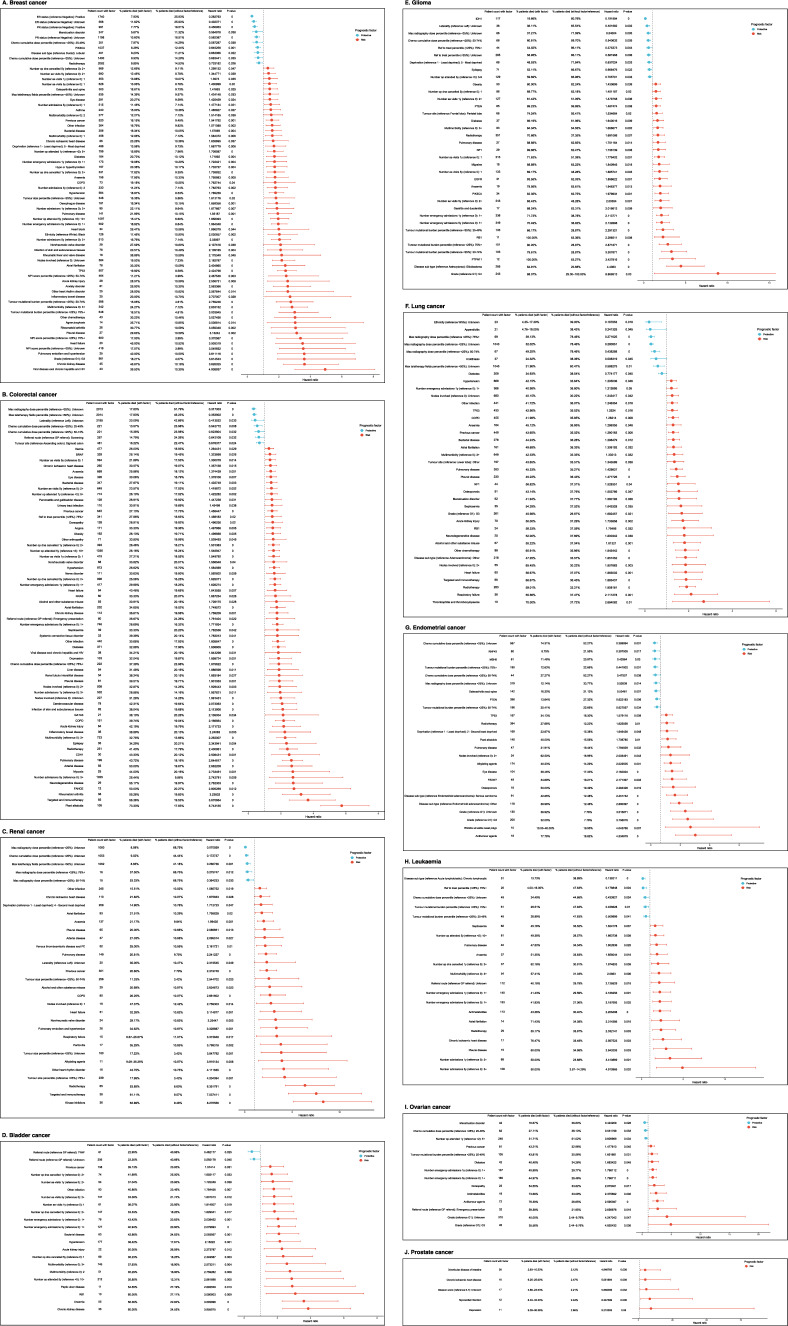
Cox proportional hazards regression results. Forest plots demonstrate significant results (p-value < 0.05) from Cox proportional regression models by cancer type. Patient counts with factor, percentages of deceased patients with factor, percentages of deceased patients without factor, hazard ratios and p-values are provided in tabular format. Hazard ratios are provided in forest plot ordered by effect size and coloured by effect direction. Term ‘risk’ is used where a factor was found to be associated with shorter overall survival, while term ‘protective’ is used where a factor was found to be associated with longer overall survival. **A** Breast cancer. Cox proportional hazards regression results for breast cancer. **B** Colorectal cancer. Cox proportional hazards regression results for colorectal cancer. **C** Renal cancer. Cox proportional hazards regression results for renal cancer. **D** Bladder cancer. Cox proportional hazards regression results for bladder cancer. **E** Glioma. Cox proportional hazards regression results for glioma. **F** Lung cancer. Cox proportional hazards regression results for lung cancer. **G** Endometrial cancer. Cox proportional hazards regression results for endometrial cancer. **H** Leukaemia. Cox proportional hazards regression results for leukaemia. **I** Ovarian cancer. Cox proportional hazards regression results for ovarian cancer. **J** Prostate cancer. Cox proportional hazards regression results for prostate cancer

Age and stage factors were found to be significant in most models with a consistent effect on overall survival. Older age and higher stage were associated with shorter survival across all cancer types. There were mixed results observed for sex variable with some models showing no significant difference for overall survival between male and female patients (e.g., some models in bladder, leukaemia and renal cancers). It could be considered to exclude sex variable in such cases in order to simplify the model. There were 116 unique factors found to have a significant prognostic effect for overall survival outcomes across all cancer types when adjusted for age, sex and stage (Fig. 7). Higher tumour grade was associated with shorter overall survival in breast, endometrial, glioma, lung and ovarian cancers. Larger or unknown number of lymph nodes involved were associated with worse overall survival outcomes in breast, colorectal, endometrial, lung and renal cancers. Prognostic effects of grade and lymph node status were consistent with the findings in previous literature [28–31].

A few medical conditions were associated with overall survival outcomes on pan-cancer level. Anaemia was associated with shorter overall survival in all cancer types except endometrial, ovarian and prostate cancers. Pulmonary disease was associated with shorter overall survival in all cancer types except bladder, ovarian and prostate cancers. Acute kidney injury, atrial fibrillation, bacterial disease, chronic ischaemic heart disease, COPD, diabetes, heart failure, hypertension, other infection, pleural disease and previous cancer were associated with reduced overall survival in four or more cancer types with one exception. Diabetes was associated with longer overall survival in lung cancer. Multimorbidity was associated with shorter overall survival in most cancer types, which was consistent with previous studies [32, 33].

Multiple factors related to previous hospital encounters were found to have a significant association with overall survival in most cancer types (all cancers except endometrial, prostate and renal), such as previous hospital admissions, A&E department visits, attended outpatient appointments and outpatient appointments that were cancelled or where patient did not attend. Generally, a larger number of previous hospital encounters was associated with worse overall survival outcomes in all cancer types with two exceptions. Larger number of attended outpatient appointments in glioma and ovarian cancers were found to be associated with longer overall survival.

A few behavioural factors were identified as significant prognostic factors in overall survival. Alcohol and other substance misuse was associated with shorter overall survival in colorectal, lung and renal cancers. Obesity was associated with shorter overall survival in glioma and colorectal cancers. Association between alcohol misuse and obesity with overall survival was also reported in previous studies [34, 35].

Higher chemotherapy cumulative dose was associated with longer overall survival in breast, colorectal, endometrial, glioma, and ovarian cancers, which could indicate successful treatment outcomes. One exception was observed in colorectal cancer where patients with chemotherapy cumulative dose in the top quartile had shorter survival compared to patients in the bottom quartile. This result might be representative of sicker patients receiving larger doses of chemotherapy. Larger or unknown maximum radiography doses were associated with longer overall survival in colorectal, endometrial, glioma, lung, and renal cancers. However, the presence of radiotherapy treatment was associated with longer overall survival only in breast cancer. The opposite effect was observed in colorectal, endometrial, glioma, leukaemia, lung, and renal cancers.

**Fig. 8:**
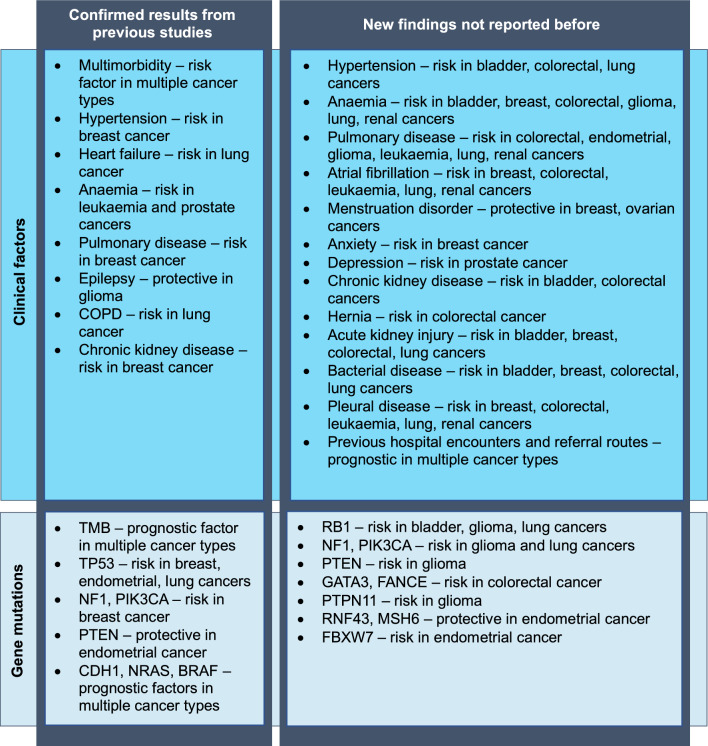
Summary of key findings. Table represents key findings of the study broken down to clinical factors and genetic mutations. The table distinguishes between findings that confirmed results reported previously and new findings that were not reported before based on the systematic review conducted by the authors [3]. Term ‘risk’ is used where a factor was found to be associated with shorter overall survival, while term ‘protective’ is used where a factor was found to be associated with longer overall survival. Term ‘prognostic’ is used where factor effect direction differed across cancer types

TMB effects on overall survival were different across cancer types. Higher TMB was associated with shorter overall survival in breast, glioma and ovarian cancers, while the opposite effect was found in leukaemia and endometrial cancers. Gene mutations in TP53 gene were associated with shorter overall survival in breast, endometrial and lung cancers. Gene mutations in RB1 gene were associated with shorter overall survival in bladder, glioma and lung cancers. Three other genes were found to be significant prognostic factors for overall survival in more than one cancer type, NF1, PIK3CA and PTEN. Somatic mutations in NF1 gene were associated with shorter survival in glioma and lung cancers. Somatic mutations in PIK3CA gene were associated with shorter survival in glioma, but had an opposite effect in breast cancer. Somatic mutations in PTEN gene were associated with shorter survival in glioma, but had an opposite effect in endometrial cancer. The same effect in endometrial cancer was reported previously [36]. A summary of key findings about prognostic factors for overall survival in cancer patients in this study are presented in Fig. 8.

## Discussion

This study explored important prognostic factors and associations using linked clinical and genetic data for ten cancer types including less commonly researched types such as glioma and bladder cancer. The analyses were conducted on a cohort of 9977 cancer patients, which is significantly larger than the average sample size in previous similar studies. 161 clinical and 124 genetic variables were defined and investigated through large scale data linking, cleaning, processing, feature engineering and statistical modelling. Of these, 116 unique factors were found to have significant prognostic effect for overall survival outcomes across ten cancer types when adjusted for age, sex and stage. The largest number of significant prognostic factors was observed in colorectal cancer (66 factors), and the smallest number was observed in prostate cancer (5 factors). The largest number of significant genetic prognostic factors for overall survival was found in glioma (8 factors).

A summary of previously published literature [3] shows that most genetic studies tend to focus on one biological pathway or group of genes associated with cancer leading to less overlap across genetic studies. The number of genes and genetic mutations investigated in this study is considerably larger than in previous studies. A selected list of 790 genes were included in this analysis and 303,748 variants were found in these genes in the cohort of 9977 patients. After application of variant filtering techniques, 99 genes were included in the descriptive and survival analyses. Analysis on such a large scale helps improve the knowledge about cancer progression, prognosis, and potential treatments for these ten cancer types.

### Key results and contribution to existing literature

Descriptive analysis showed large differences in overall survival outcomes across cancer types. The most advanced tumours were found in glioma, colorectal and ovarian cancers, while the least advanced tumours were found in prostate, breast, and endometrial cancers. The greatest differences in demographic characteristics were identified for patient’s age. Patients with glioma and leukaemia were generally younger, while patients with bladder, colorectal and lung cancers were older. Older patients had lower survival probabilities in all cancer types compared to younger patients. Similar effects of age on overall survival in cancer were reported in previous studies [37, 38].

Hypertension was the most common pre-existing condition across all cancer types and was found to be a significant prognostic factor in four cancer types, including bladder, breast, colorectal and lung cancers, which has been observed in previous studies [39]. Other heart-related conditions were most prevalent in bladder and lung cancers with heart failure being a prognostic risk factor for overall survival in lung cancer, which has been reported before [40]. Atrial fibrillation was found to be a significant prognostic factor for overall survival in breast, colorectal, leukaemia, lung and renal cancers, which has not been reported before. Anaemia and pulmonary disease were common pre-existing conditions with significant association with shorter overall survival in seven cancer types. Association between anaemia and overall survival was reported in leukaemia and prostate cancers in previous studies [11, 41], but this study showed insignificant results for prostate cancer. In this patient cohort, only 3.7% of prostate cancer patients had anaemia, which could explain the insignificant results. Similarly, association between pulmonary disease and overall survival was reported in breast cancer before [39].

Female health-related conditions were prevalent in breast, endometrial and ovarian cancers with menstruation disorder identified as a protective prognostic factor for overall survival in breast and ovarian cancers. Anxiety was identified as a risk factor for breast cancer prognosis and depression as a risk factor for prostate cancer prognosis. Association between mental health and female health-related conditions with overall survival in cancer was not reported before. Acute kidney injury, bacterial disease and pleural disease were among conditions not reported to have association with overall survival before, while in this analysis identified as significant prognostic risk factors in four or more cancer types.

Some conditions were prevalent in specific cancer types such as epilepsy in glioma (protective factor for overall survival, which has been reported before [29]), COPD in lung cancer (risk factor for overall survival, which has been reported before [40]), chronic kidney disease in bladder and renal cancers (risk factor for overall survival in bladder cancer), hernia in bladder, colorectal, lung and renal cancers (risk factor for overall survival in colorectal cancer). Generally, multimorbidity, history of previous cancer and history of previous hospital encounters were associated with shorter overall survival in most cancer types. Association between previous hospital encounters and cancer survival has not been well explored before, however this analysis showed that previous hospital admissions, A&E department visits and outpatient appointments are important prognostic factors. The analysis of referral routes demonstrated protective effects of cancer screening in colorectal cancer and risk effects of referral through emergency presentation in ovarian cancer.

Considerable differences in treatment strategies were identified when comparing cancer types. Radiotherapy and chemotherapy using alkylating agents were the most popular treatment types at pan-cancer level, however the use of these treatments varied across cancer types. About 80% of patients with glioma and breast cancers had radiotherapy, while less than 10% of renal and ovarian cancer patients had radiotherapy. Antimetabolites treatment was more common in leukaemia and colorectal cancers than any other treatment types. Hormone therapy was common in breast and prostate cancers but was rarely used in other cancer types. Mixed prognostic effects of treatments for overall survival were observed in Cox proportional hazards regression analysis. Larger chemotherapy and radiography doses were generally associated with longer overall survival in most cancer types, but the presence of some treatment types was associated with shorter overall survival. Clinical treatment choices strongly depend on cancer progression at the point of diagnosis, measured by stage and grade. Without more advanced multivariable analysis considering these interactions or a causal inference analysis, it could not be concluded whether certain therapy associations with shorter survival show ineffective treatment strategies or that those therapies are generally used for patients with more advanced cancers.

The highest tumour mutational burden as well as the highest variation in TMB were observed in lung, colorectal and bladder cancers, while the lowest TMB was in patients with leukaemia, prostate, and breast cancers. TMB effects on overall survival were different across cancer types with protective effects observed in leukaemia and endometrial cancers and risk effects observed in breast, glioma, and ovarian cancers. Association between TMB and cancer outcomes was reported before [42]. Germline variants were very rare and the only two genes with more than 2% prevalence in the patient cohort where BRCA1 and BRCA2, which was identified in ovarian cancer. No germline variants were found to be associated with overall survival when adjusted for age, sex and stage.

TP53 and PIK3CA were genes with the largest number of somatic variants across multiple cancer types. Although mutations in gene TP53 were found to be the most common of all genes in patients with ovarian cancer, overall frequency of 38% was lower than near to 100% frequencies previously reported for high-grade serous ovarian cancer [43]. This could be explained by inclusion of other types of ovarian cancer as well as a large proportion of patients with lower stage and grade in our patient cohort. Somatic mutations in genes TP53 and RB1 were associated with shorter overall survival after adjusting for age, sex, and stage in three cancer types. These results were comparable to previous studies [15, 44–46], however this analysis found mutations in RB1 gene to be associated with overall survival in bladder, glioma and lung cancers that was not reported before. Three other genes were found to be significant prognostic factors for overall survival in more than one cancer type; NF1, PIK3CA and PTEN. NF1 and PIK3CA gene mutations were reported to have a risk prognostic effect in breast cancer [47], while this analysis also showed association in glioma and lung cancers. PTEN gene was known to be associated with overall survival in colorectal and endometrial cancers [36, 48], but this analysis also showed association in glioma. Mutations in genes CDH1, NRAS and BRAF were known to be associated with overall survival from previous studies [48–51] which was also confirmed in this analysis. Among previously not reported associations with overall survival were mutations in GATA3 and FANCE genes in colorectal cancer, mutations in gene PTPN11 in glioma, and mutations in RNF43, MSH6 and FBXW7 genes in endometrial cancer.

### Limitations of data and methodology

Several limitations were recognised in the data used in this study. The majority of genetic samples collected from the participants of the 100,000 Genomes Project were taken from surgical resection showing that most patients in the cohort had surgical treatment. Therefore, results based on this data are representative of cancer patients who undergo surgery and might not be generalisable to all cancer patients. Another limitation of the data is the difference in sample sizes across cancer types. There were more patients in the cohort with more common cancer types such as breast, colorectal and lung cancer, while fewer patients for less common cancer types such as leukaemia, glioma, bladder and ovarian cancer. All analyses in this study were conducted by splitting the data by cancer type, therefore results are not driven by the most common cancer types. However, having larger samples for less common cancer types could help identify more prognostic factors related to overall survival.

Although multiple rich data sources were linked to obtain a comprehensive cancer dataset in this study, other data sources such as General Practice (GP) data could add value to this analysis. For example, certain behavioural factors such as smoking, alcohol use, body mass index (BMI) and certain long-term conditions are better recorded in GP data. Finally, a high level of data missingness was observed for some variables such as tumour size, referral route, waiting times, chemotherapy dose, and radiography dose.

More advanced multivariable analyses are needed to investigate prognostic effects of factors for overall survival considering potential confounding factors, multicollinearity and interactions between factors, which will be the focus of our future work in this area.

## Conclusions

Despite the advancement of the use of clinical and genetic data for personalised cancer treatments in the recent years, there is still a gap between the research findings on prognostic biomarkers and their use in clinical practice. Few prognostic biomarkers have been successfully translated from discovery to clinical adoption to date requiring more comprehensive and clear information about the link between prognostic factors and patient outcomes [52].

This study provides a unique analysis of clinicopathological and genetic prognostic factors across ten cancer types and a large patient sample using the extensive dataset from the participants of Genomics England’s 100,000 Genomes Project. The findings confirmed that well known factors such as age, tumour stage, size and grade, comorbidities, referral route, waiting times can be used to predict cancer survival. Many of these factors are already widely used in clinical practice for determining prognosis and choosing the treatment of cancer. However, this study brings to light other, less commonly recognised, factors which might help improve the precision when determining cancer prognosis and survival. Among clinicopathological factors that could be used for prognosis prediction are comorbidities such as anaemia, pulmonary disease, kidney disease and COPD, lifestyle factors such as alcohol use and obesity, and history of previous hospital encounters. This work demonstrates the prognostic ability of tumour mutational burden and somatic mutations in well identified genes such as TP53 as well as less commonly recognised genes such as RB1, NF1, PIK3CA and PTEN.

The results from this study bring significant contributions to the fast-growing knowledge base about cancer disease and could be used by researchers to further explore the prognostic power of clinical and genetic factors in cancer survival. The information about important factors could be used to build prognostic models which could help improve the accuracy of cancer prognosis leading to improvements in patient outcomes.

## Supplementary Information

Additional file1

Additional file2

Additional file3

Additional file4

Additional file5

## Data Availability

The data that support the findings of this study are available in Genomics England’s cloud-based workspace called Research Environment, but restrictions apply to the access of these data. To access the data, researchers must first apply to become a member of the Genomics England Clinical Interpretation Partnership (GECIP). R code used to generate data analyses and statistical analyses are available from the corresponding author on reasonable request.

## Abbreviations

BMI: Body mass index
COPD: Chronic obstructive pulmonary disease
GECIP: Genomics England Clinical Interpretation Partnership
GMC: Genomics Medicine Centre
GP: General practice
HES: Hospital Episode Statistics
ICD10: 10th revision of the International Classification of Diseases
IMD: Index of Multiple Deprivation
NCRAS: National Cancer Registration and Analysis Service
ONS: Office of National Statistics
OPCS: Operating Procedure Codes Supplement
RNA: Ribonucleic acid
SNV: Single nucleotide variant
TMB: Tumour mutational burden
