# Supplementary material for: Prognostic determinants in cancer survival: a multidimensional evaluation of clinical and genetic factors across 10 cancer types in the participants of Genomics England’s 100,000 Genomes Project
Source: Discov Oncol. 2024 Sep 15;15:448. doi: 10.1007/s12672-024-01310-8 (PMC11402888; doi:10.1007/s12672-024-01310-8)

Percentage of patients with a tumour in each site out of total patients by cancer type. Percentages sum up to 100% for each cancer type. Box sizes represent the patient counts in the whole patient cohort. For cancers that did not have classification to different sites, one box with 100% is included.

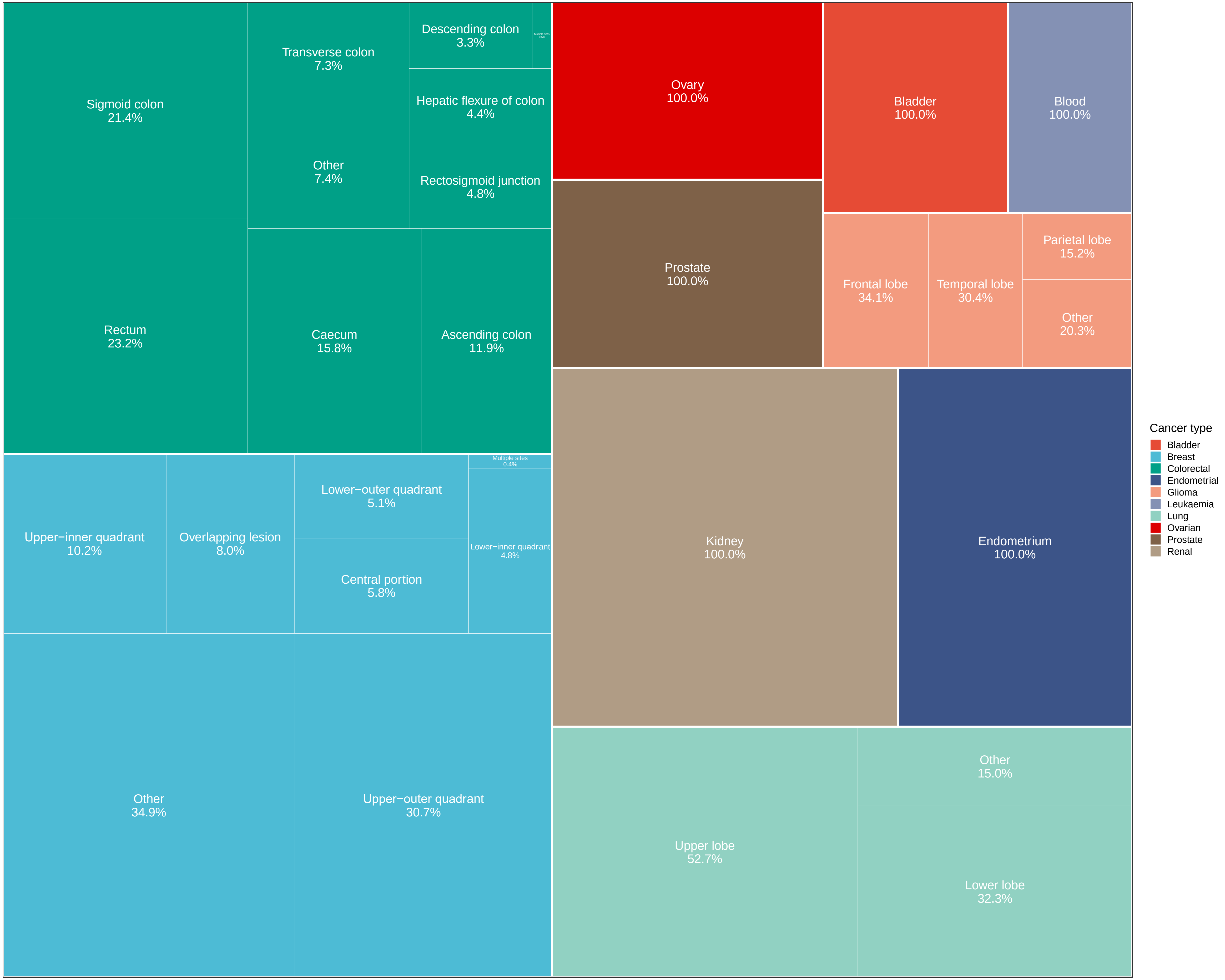

Supplement: Supplementary file 1 — Additional file1 [file 12672_2024_1310_MOESM1_ESM.pdf]
