# Supplementary material for: Prognostic determinants in cancer survival: a multidimensional evaluation of clinical and genetic factors across 10 cancer types in the participants of Genomics England’s 100,000 Genomes Project
Source: Discov Oncol. 2024 Sep 15;15:448. doi: 10.1007/s12672-024-01310-8 (PMC11402888; doi:10.1007/s12672-024-01310-8)

(A) Chemotherapy cumulative dose. Violin plot demonstrates distribution of cumulative chemotherapy dose by cancer type. (B) Maximum radiography dose. Violin plot demonstrates distribution of maximum radiography dose by cancer type. (C) Maximum teletherapy fields. Violin plot demonstrates distribution of maximum teletherapy fields by cancer type.

A. Chemotherapy cumulative dose

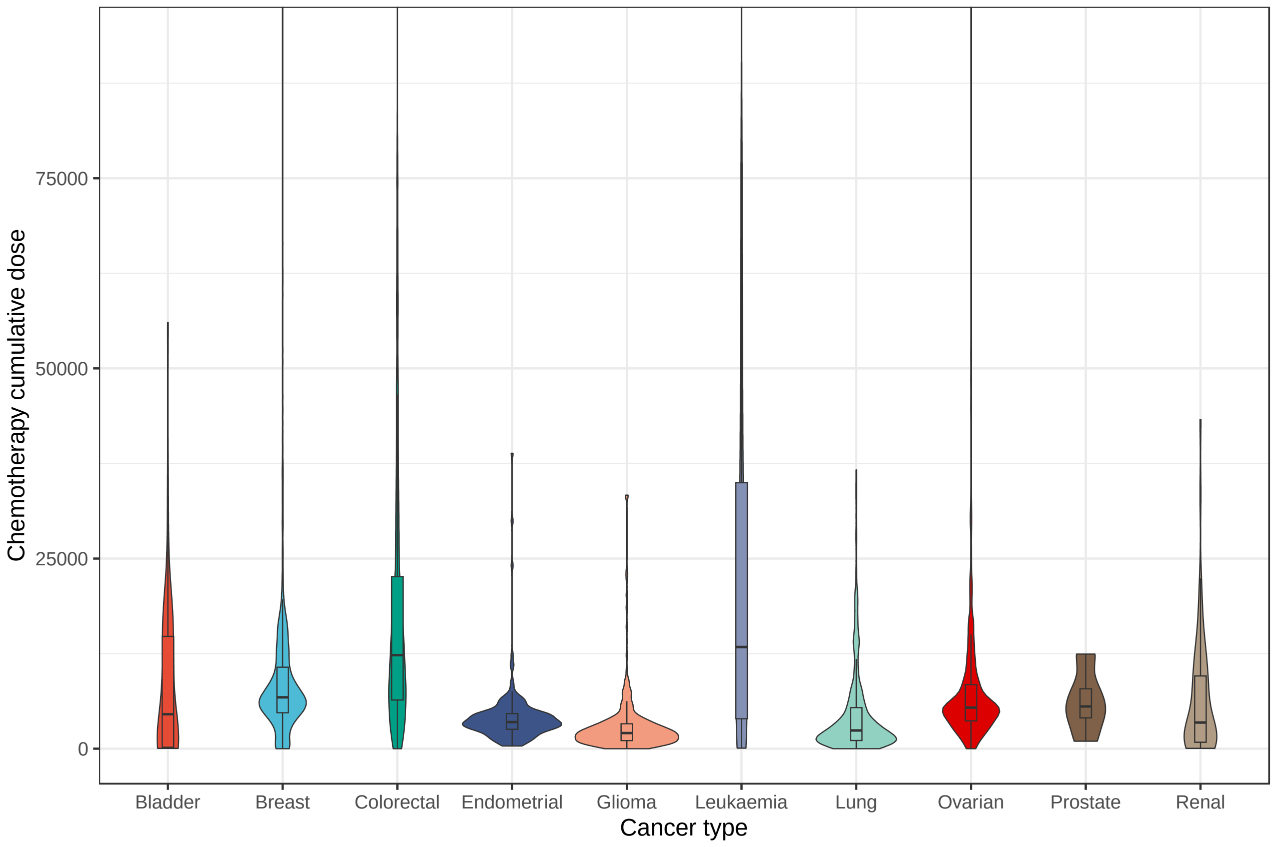

B. Maximum radiography dose

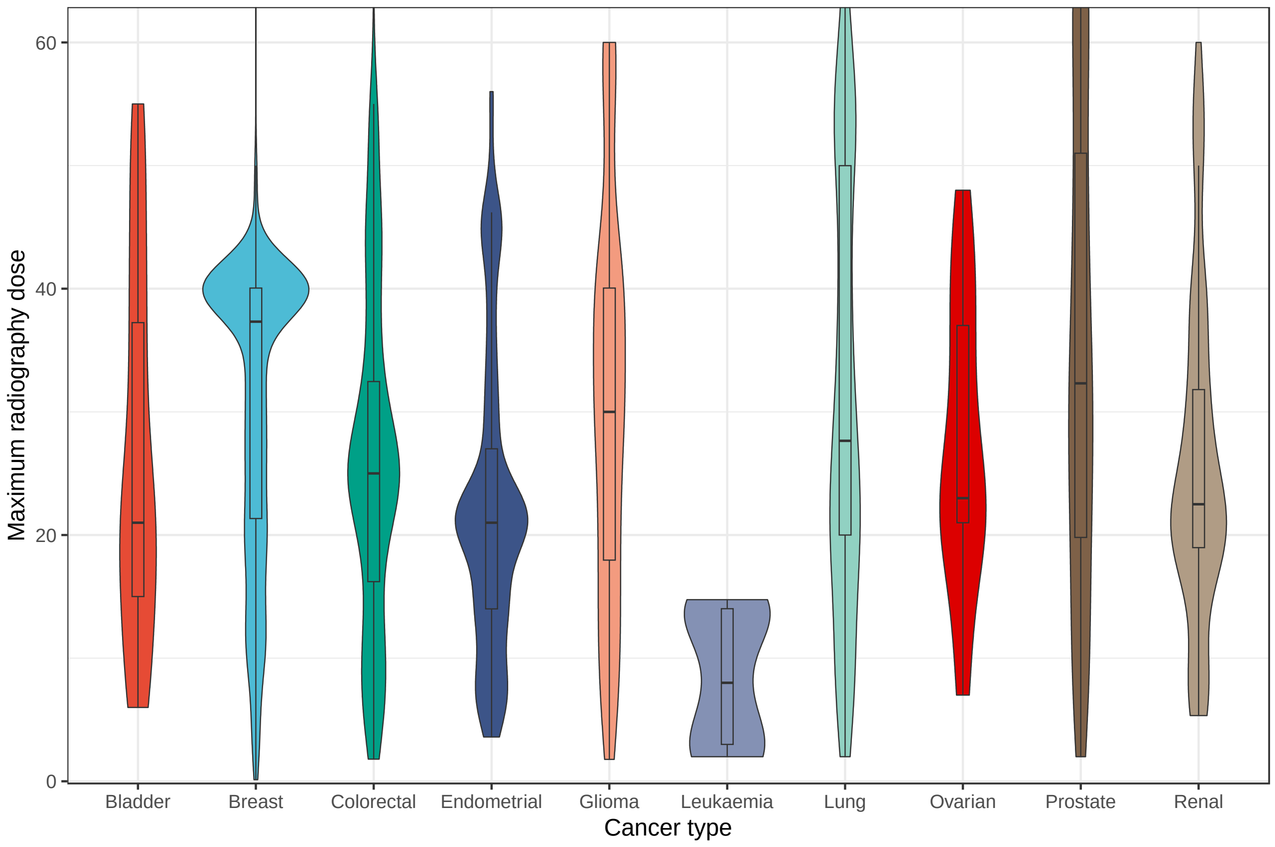

C. Maximum teletherapy fields

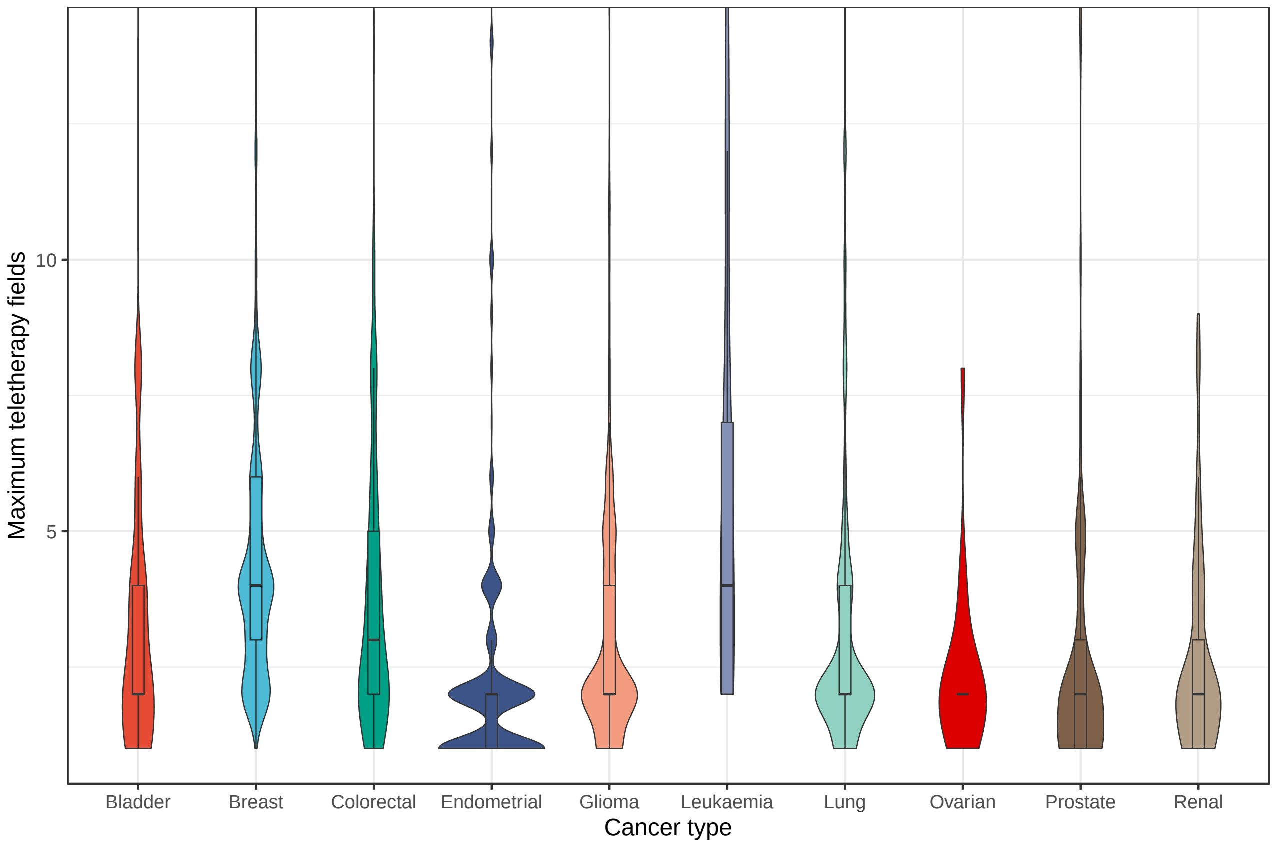

Supplement: Supplementary file 2 — Additional file2 [file 12672_2024_1310_MOESM2_ESM.pdf]
