# Supplementary material for: Prognostic determinants in cancer survival: a multidimensional evaluation of clinical and genetic factors across 10 cancer types in the participants of Genomics England’s 100,000 Genomes Project
Source: Discov Oncol. 2024 Sep 15;15:448. doi: 10.1007/s12672-024-01310-8 (PMC11402888; doi:10.1007/s12672-024-01310-8)

(A) Mean number of admissions (all types). Bar chart demonstrates mean number of admissions (all types) over different time periods by cancer type. (B) Mean number of emergency admissions. Bar chart demonstrates mean number of emergency admissions over different time periods by cancer type.

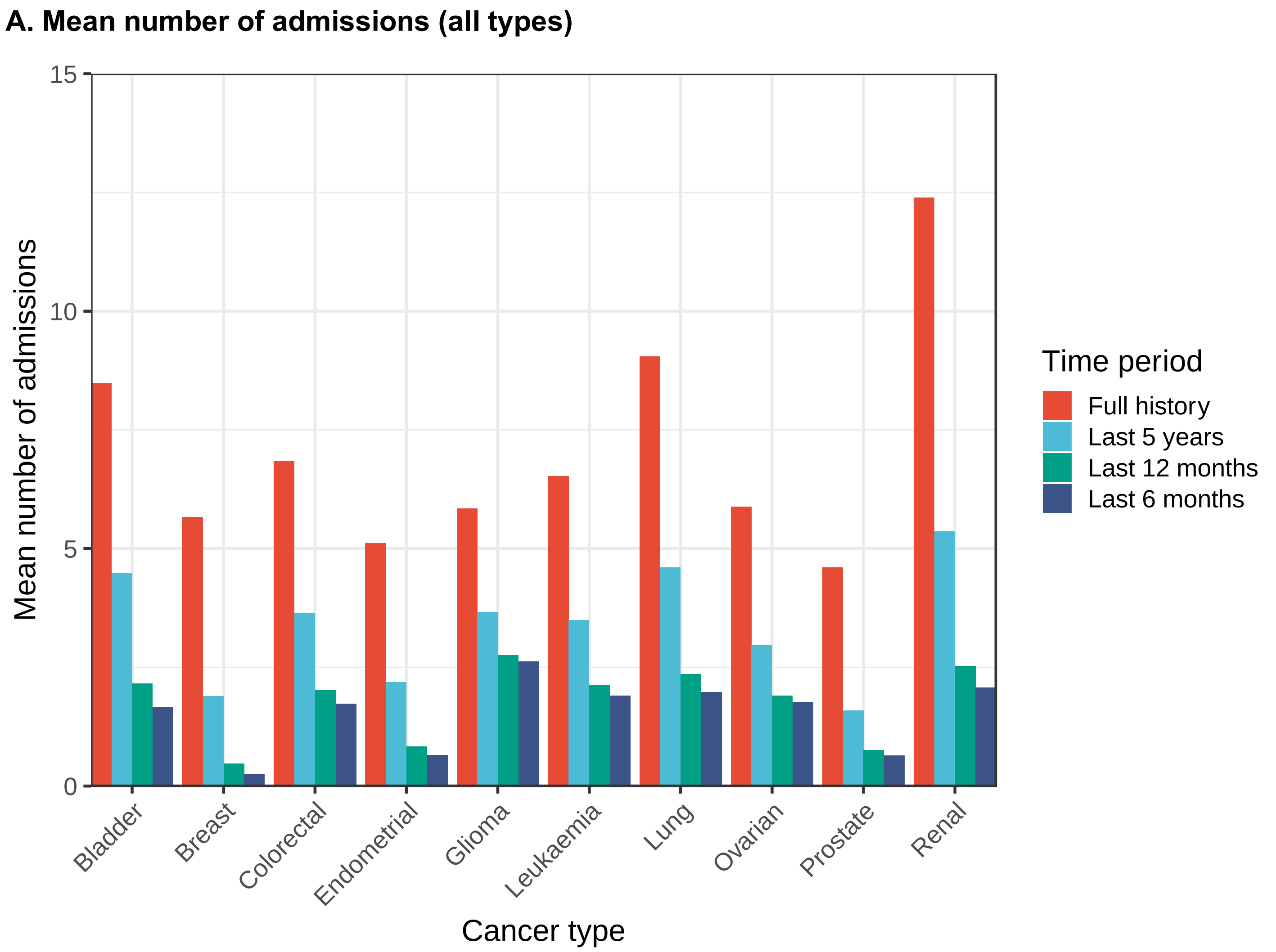

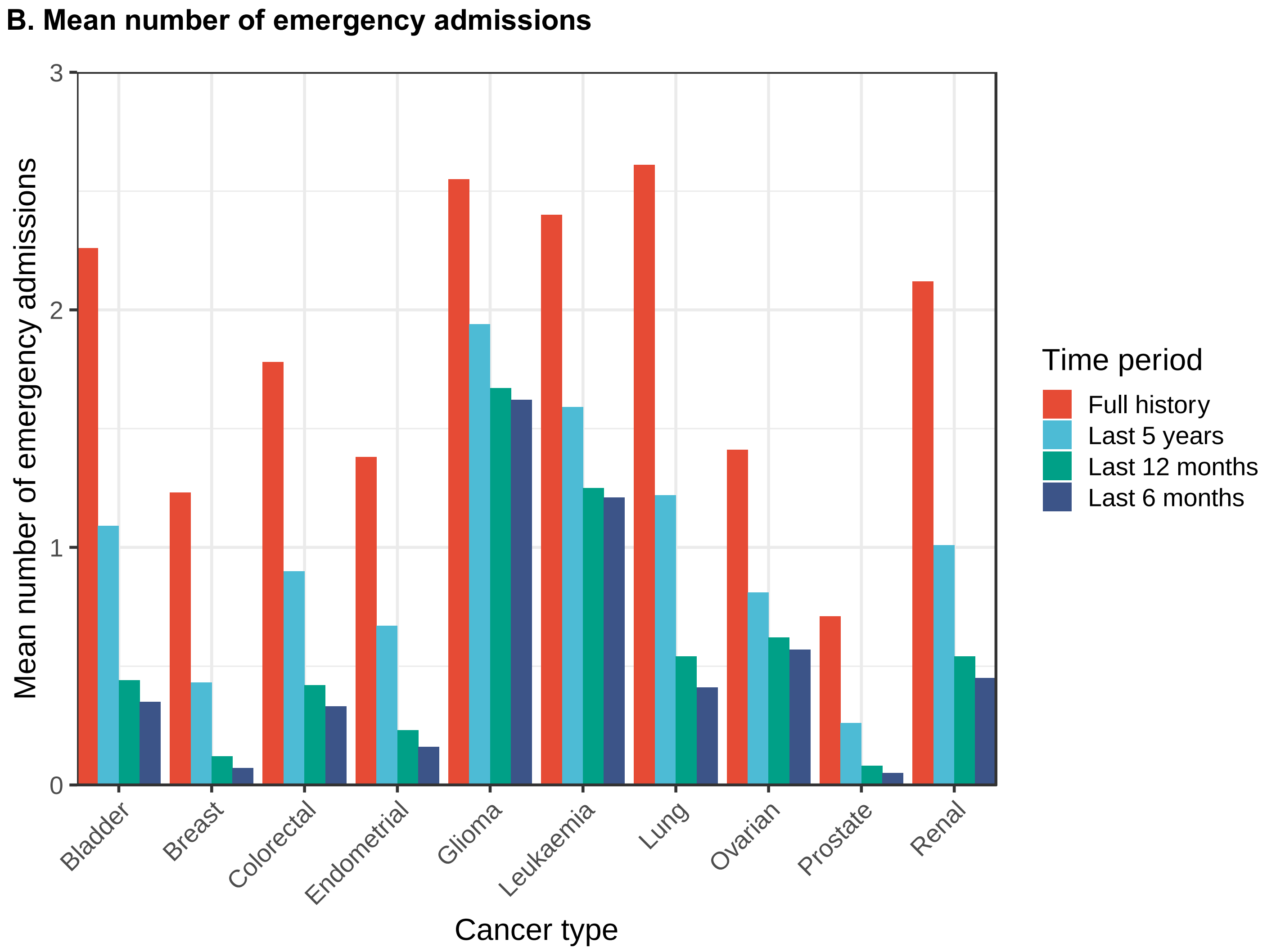

Supplement: Supplementary file 3 — Additional file3 [file 12672_2024_1310_MOESM3_ESM.pdf]
