# Supplementary material for: Prognostic determinants in cancer survival: a multidimensional evaluation of clinical and genetic factors across 10 cancer types in the participants of Genomics England’s 100,000 Genomes Project
Source: Discov Oncol. 2024 Sep 15;15:448. doi: 10.1007/s12672-024-01310-8 (PMC11402888; doi:10.1007/s12672-024-01310-8)

Heatmap demonstrates frequencies of germline mutations in selected genes compared across cancer types. Frequencies are calculated as percentage of patients who had the mutation out of total patients in cancer type. To avoid patient identification, percentages lower than 2% that could imply small patient counts were combined into one category and presented as “<2.0%”.

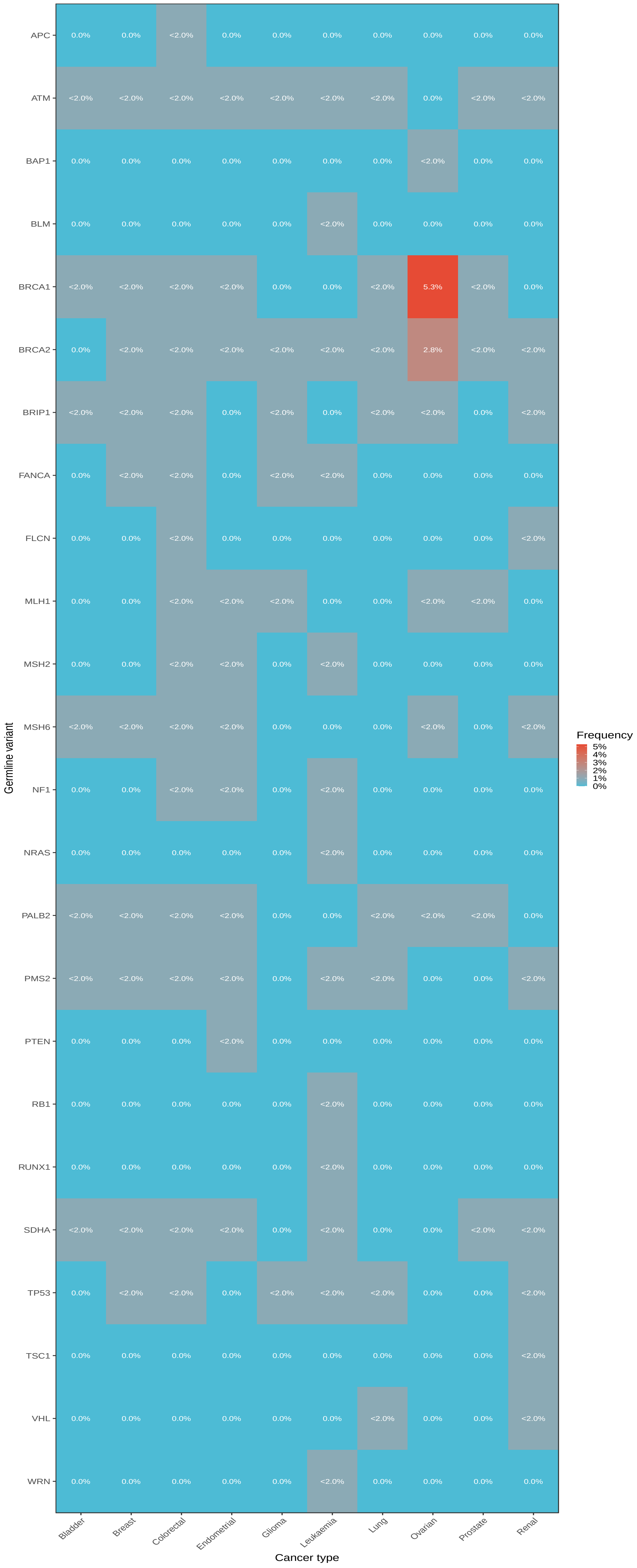

Supplement: Supplementary file 4 — Additional file4 [file 12672_2024_1310_MOESM4_ESM.pdf]
