# Supplementary material for: Prognostic determinants in cancer survival: a multidimensional evaluation of clinical and genetic factors across 10 cancer types in the participants of Genomics England’s 100,000 Genomes Project
Source: Discov Oncol. 2024 Sep 15;15:448. doi: 10.1007/s12672-024-01310-8 (PMC11402888; doi:10.1007/s12672-024-01310-8)
Supplement: Supplementary file 5 — Additional file5 [file 12672_2024_1310_MOESM5_ESM.pdf]

Kaplan Meier survival curves demonstrate disease specific survival probability over 5 years by cancer type. Numbers at risk demonstrate patient counts with percentage of total patients in cancer type provided in brackets.

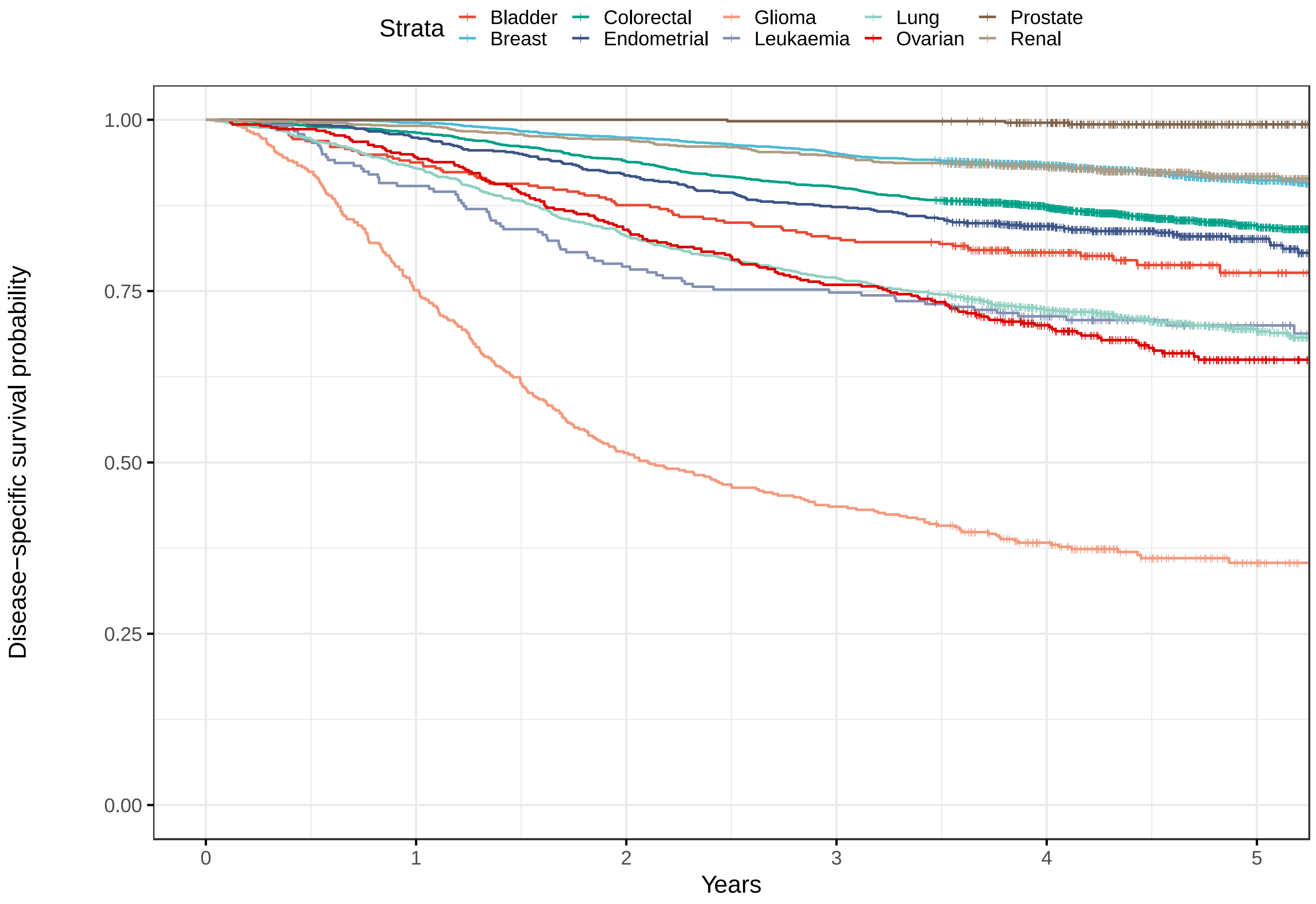

Number at risk: n (%)

Strata	0	1	2	3	4	5
Bladder	353 (100)	331 (94)	309 (88)	292 (83)	190 (54)	49 (14)
Breast	2605 (100)	2594 (100)	2537 (97)	2478 (95)	2007 (77)	902 (35)
Colorectal	2247 (100)	2204 (98)	2110 (94)	2026 (90)	1609 (72)	662 (29)
Endometrial	762 (100)	742 (97)	700 (92)	665 (87)	518 (68)	190 (25)
Glioma	434 (100)	326 (75)	223 (51)	189 (44)	130 (30)	44 (10)
Leukaemia	238 (100)	215 (90)	187 (79)	178 (75)	137 (58)	69 (29)
Lung	1317 (100)	1224 (93)	1094 (83)	1013 (77)	799 (61)	351 (27)
Ovarian	436 (100)	412 (94)	366 (84)	331 (76)	248 (57)	105 (24)
Prostate	462 (100)	462 (100)	462 (100)	461 (100)	431 (93)	252 (55)
Renal	1123 (100)	1113 (99)	1090 (97)	1063 (95)	862 (77)	322 (29)

Years
